# Complete genome sequence of a *Capillovirus* infecting *Citrus medica* L. in China

**DOI:** 10.1128/mra.00565-25

**Published:** 2025-11-05

**Authors:** Yaqin Wang, Yuheng Zhang, Shi Wu, Xiaojun Zhou, Xiaochan He, Xueping Zhou, Zhanqi Wang, Liyan Zhu

**Affiliations:** 1 State Key Laboratory of Rice Biology and Breeding, Institute of Biotechnology, Zhejiang University 12377, Hangzhou, China; 2 Jinhua Academy of Agricultural Sciences, Jinhua, China; 3 Zhejiang Provincial Key Laboratory of Biology of Crop Pathogens and Insects, College of Life Sciences, Huzhou University 117774, Huzhou, China; Katholieke Universiteit Leuven, Leuven, Belgium

**Keywords:** *Citrus medica*, *Capillovirus*, apple stem grooving virus, complete sequence

## Abstract

Apple stem grooving virus (ASGV) can infect various fruit and herbaceous plants. Here, we report an isolate of ASGV from *Citrus medica* in Jinhua City, Zhejiang Province, China, and reveal that it belongs to the genus *Capillovirus* within the family *Betaflexiviridae*.

## ANNOUNCEMENT

Apple stem grooving virus (ASGV), also known as citrus tatter leaf virus (CTLV), is a member of the species *Capillovirus mali* that belongs to the genus *Capillovirus* within the family *Betaflexiviridae* (1, 2). It can elicit severe symptoms in natural hosts, such as stem grooving, necrosis at grafting sites, leaf deformation, chlorotic spots, and ring spots (1–3). ASGV has a positive-sense, single-stranded RNA genome of approximately 6,500 nucleotides (nt) and comprises two overlapping open reading frames (ORFs) (4–6).

**Fig 1:**
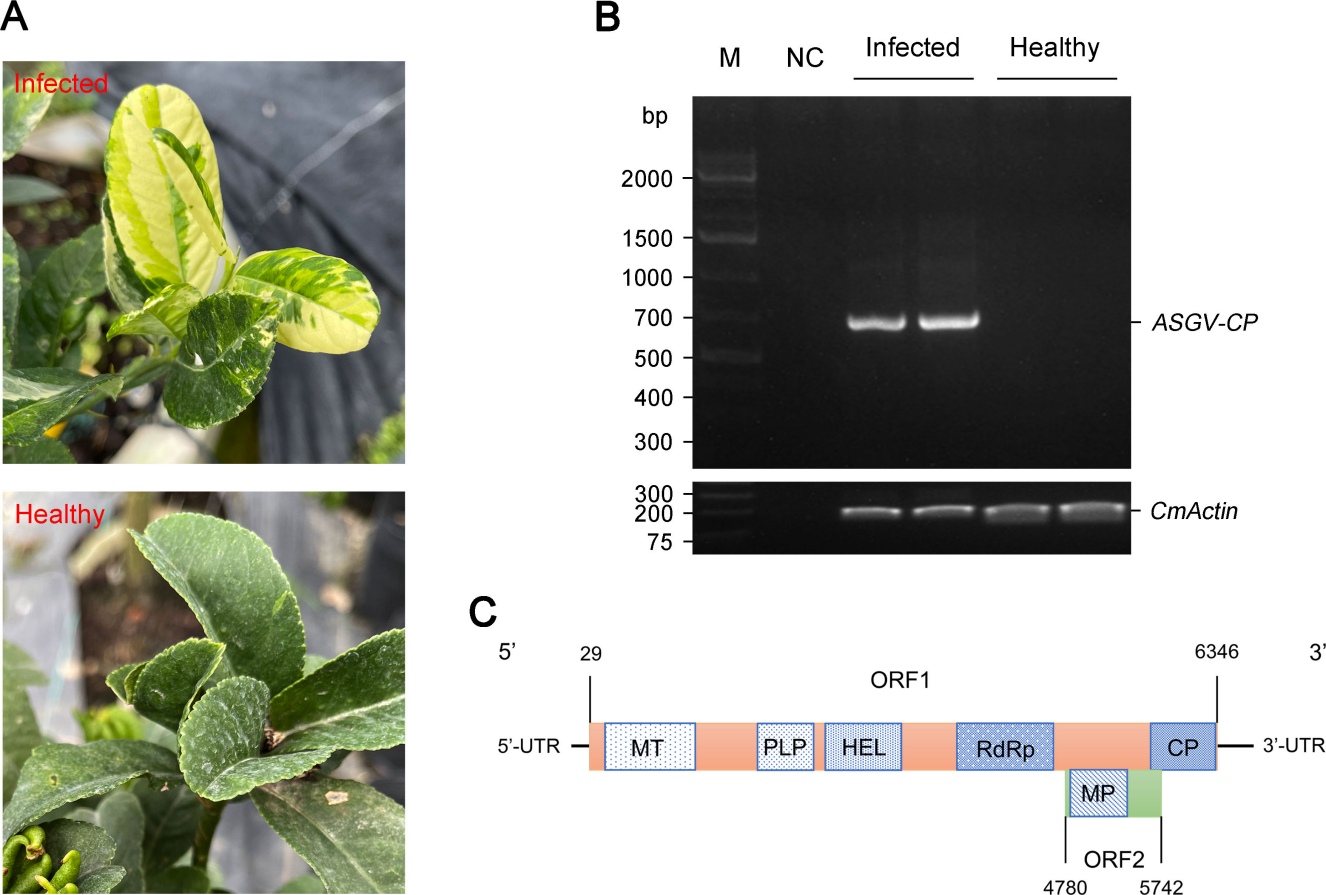
An isolate of the ASGV-JH from Jinhua City, Zhejiang Province, China, was detected in fingered citron (*Citrus medica* L.). (**A**) Virus-like symptoms of *C. medica* plants. (**B**) RT-PCR analysis of *C. medica* samples using ASGV-specific primer pairs. M, marker; NC, negative control. (**C**) Genome structure of ASGV-JH. Different rectangles indicate the putative ORFs. MT, methyltransferase; PLP, papain-like protease; HEL, helicase; RdRp, RNA-dependent RNA polymerase; CP, coat protein; and MP, movement protein.

In July 2024, *C. medica* plants that displayed chlorosis and mottling symptoms were collected in a nursery of the Jinhua Academy of Agricultural Sciences, Jinhua, Zhejiang Province, China (Fig. 1A). To identify potential viral pathogens responsible for the observed symptoms, symptomatic leaf samples were collected, and total RNA was extracted using TRIzol reagent (Invitrogen, USA). The quantity of RNA was determined using a Nanodrop Spectrophotometer (Thermo Scientific, USA), and RNA samples with an RNA integrity number > 7.0 were used for cDNA library construction. The cDNA library was prepared using the TruSeq RNA Sample Prep Kit (Illumina, USA) following the depletion of ribosomal RNA with the Ribo-Zero rRNA Removal Kit (Epicentre, USA) (7, 8). RNA sequencing was performed on the Illumina NovaSeq 6000 platform (Beijing Tangtang Biotechnology Co., Ltd., China) using a paired-end 150 bp configuration (7, 8). As a result, a total of 91,452,628 raw reads were obtained. After removing the adapter and low-quality sequences using Trimmomatic software (version 0.39) with default settings, 88,151,614 clean reads were retained and subsequently reassembled into contigs using Trinity (version 2.15.0) with default parameters. A total of 394,028 contigs were generated, with an *N*_50_ length of 1,509 nt. The assembled contigs were subsequently annotated using the BLASTn algorithm against the NCBI viral genomes database (https://www.ncbi.nlm.nih.gov/datasets/genome/). As a result, 19,847 contigs were annotated as virus-related gene fragments with an *E*-value ≤ 1 × 10⁻^6^. Among these, eight contigs exhibited high sequence similarity to the ASGVs of the genus *Capillovirus* in the family *Betaflexiviridae*, covering 99.4% of the complete genome sequence. Therefore, the tentative name “ASGV Jinhua isolate” (ASGV-JH) is proposed.

To confirm the RNA sequencing data, we performed an RT-PCR analysis using the ASGV-specific primer pairs (Table 1). As shown in Fig. 1B, ASGV-JH was successfully detected in *C. medica*, but it was not detected in healthy plants. To obtain the full-length genomic sequence of the ASGV-JH, a segmental cloning approach involving six fragments was performed using RT-PCR and 3′/5′-RACE (rapid amplification of cDNA ends, Clontech, Code No. 634858) techniques coupled with Sanger sequencing, using the primer pairs listed in Table 1. The results showed that the complete genome of ASGV-JH comprised 6,515 nt, including a 27-nt poly(A) tail, and had a GC content of 40.92%. Comparative analysis revealed that it exhibited the highest nucleotide sequence similarity of 94.6% with CTLV-TL (GenBank accession number MZ330115). ORFfinder prediction showed that the ASGV-JH genome consisted of two overlapping ORFs, with the 5′-untranslated region (5′-UTR) and the 3′-UTR measuring 28 and 142 nt in length, respectively (Fig. 1C). ORF1 (from 29 to 6,346) encodes a polyprotein with a molecular weight of 241.7 kDa, whereas ORF2 (from 4,780 to 5,742) encodes a movement protein with a molecular weight of 36.2 kDa (Fig. 1C).

**TABLE 1:** List of primers used in this study

Primer name	Primer sequence	Purpose
ASGV-F	5′-ATGAGTTTGGAAGACGTGCTT-3′	ASGV detection
ASGV-R	5′-CCCTTTTTGTCCTTCAGTACG-3′	
Actin-F	5′-CAGACCGTATGAGCAAGGAA-3′	Internal control
Actin-R	5′-GCTTAGGGATGCGAGGATAG-3′	
UPM	5′-CTAATACGACTCACTATAGGGC-3′	3′/5′-RACE
ASGV-5PF	5′-CAGCGCTTAATTTCCGCGCATTACGTCAATG-3′	5′-RACE
ASGV-5PR	5′-CACTGCTGAAGCTGCGTTTG-3′	
ASGV-F1F	5′-TTTGATAGGGGGAGGGCCTG-3′	F1 amplification
ASGV-F1R	5′-GCCATCTCTTTGAAATCAGAAG-3′	
ASGV-F2F	5′-CAATTTCTGCACATCTTGGG-3′	F2 amplification
ASGV-F2R	5′-TTCTGGTTGGCATGTTTATCAG-3′	
ASGV-F3F	5′-TTTGGACAAGACACATGAAATAG-3′	F3 amplification
ASGV-F3R	5′-GCTAGAATCACGTGGTCTTGG-3′	
ASGV-F4F	5′-TGCTTTCCTGAGGAGTTGTGG-3′	F4 amplification
ASGV-F4R	5′-GATACACTCCTACCCGGTGG-3′	
ASGV-3PF	5′-ATGAGTTTGGAAGACGTGCTT-3′	3′-RACE
ASGV-3PR	5′-TGAGAGGACAAACTCTAGACTCTAGAAAAACC-3′	

## Data Availability

The genome sequence of ASGV-JH has been deposited in GenBank under the accession number PQ824702. The raw sequencing reads have been deposited in the Sequence Read Archive (SRA) under BioProject accession number PRJNA1267124.
